# Current State of *Salmonella*, *Campylobacter* and *Listeria* in the Food Chain across the Arab Countries: A Descriptive Review

**DOI:** 10.3390/foods10102369

**Published:** 2021-10-05

**Authors:** Ihab Habib, Mohamed-Yousif Ibrahim Mohamed, Mushtaq Khan

**Affiliations:** 1 Department of Veterinary Medicine, College of Agriculture and Veterinary Medicine, United Arab Emirates University, Al Ain P.O. Box 1555, United Arab Emirates; mohamed-yousif-i@uaeu.ac.ae; 2 Department of Environmental Health, High Institute of Public Health, Alexandria University, Alexandria P.O. Box 21221, Egypt; 3 School of Veterinary Medicine, Murdoch University, Perth 6150, Australia; 4 Department of Microbiology and Immunology, College of Medicine and Health Sciences, United Arab Emirates University, Al Ain P.O. Box 17666, United Arab Emirates

**Keywords:** foodborne infection, Middle East, zoonoses, food safety, one health

## Abstract

Foodborne infections caused by bacterial pathogens are a common cause of human illness in the Middle East, with a substantial burden of economic loss and public health consequences. This review aims at elucidating recent literature on the prevalence of Non-Typhoidal *Salmonella* (NTS), *Campylobacter* and *Listeria monocytogens* in the food chain in the Arab countries, and to consolidate available evidence on the public health burden and the status of antimicrobial resistance (AMR) among the concerned three pathogens. The reviewed evidence points to a scarcity of understanding of the magnitude of NTS in the food chain in the Arab countries. Additionally, not much work has been done at the molecular characterization level to address the source-attribution of NTS in the Arab World. Very few surveys have been done on *Campylobacter* in the food chain in the Gulf Cooperation Council (GCC) countries. There is a gap in quantitative (counts/numbers) surveillance efforts for *Campylobacter* in the chicken meat supply across all Arab countries, despite the availability of some qualitative (presence/absence) surveillance data. While there are several reports on *L. monocytogenes* in animal-sourced foods, notably in North African Arab countries, fewer are published on *L. monocytogenes* in plant-sourced foods. Information on the *L. monocytogenes* serotypes and strain diversity circulating in the Arab region is widely lacking. Antibiotic resistance in the three pathogens is not fully understood across the Arab region, despite some reports indicating varying trends at the human–food interface. The literature evidence presented in this review stresses that *Salmonella*, *Campylobacter* and *L. monocytogenes* continue to challenge food safety and public health in the Arab countries.

## 1. Introduction

Globally, foodborne pathogens continue to be a significant challenge to food safety and international trade. In Europe and many other countries, foodborne infection is a major public health concern [1]. According to the European Centre for Disease Prevention and Control (ECDC) and the Zoonoses Reports of the European Food Safety Authority (EFSA), the incidence of human campylobacteriosis (predominantly caused by *Campylobacter jejuni*) surpassed salmonellosis in several incidences over the last ten years [2,3]. In the United States of America (USA), *Campylobacter jejuni* has been reported as the most common pathogen causing foodborne illnesses, followed by *Listeria* and *Salmonella* and the Foodborne Diseases Active Surveillance Network (FoodNet) of the Centre for Disease Control (CDC) reported that the incidence of foodborne attributed hospitalization caused by campylobacteriosis and salmonellosis was increasing over the years [4].

According to the World Health Organization (WHO) [5], the Middle East and North Africa (MENA) region reported the third-highest estimated burden of foodborne disease per population and came just after the African and the South-East Asian regions. This study of the WHO also estimated that 70% of the MENA region’s foodborne disease burden is caused by *E. coli*, non-typhoidal *Salmonella* (NTS), *Campylobacter* and Norovirus [5], stressing the severe burden caused by pathogens involved in causing foodborne infections. In the Middle East, the true incidence of foodborne infections is difficult to determine since there are limited systematic surveillance efforts to detect sporadic cases and outbreaks and to provide isolates that could be used for source-attribution and risk assessment at national and regional levels. In addition to the lack of reliable data on the occurrences of pathogens and infections, the situation of antimicrobial resistance in bacteria causing foodborne infections is not fully understood across the Middle East region, despite some reports indicating varying trends at the human–food interface [6,7,8].

In this review, we collate updated knowledge on the epidemiology of three important pathogens commonly causing foodborne infections in humans in the Arab World, and globally, i.e., Non-Typhoidal *Salmonella*, *Campylobacter* and *Listeria*. This study was undertaken using the descriptive literature review method. A descriptive review is a planned technique of locating, gathering and appraising a collection of writings on a particular research question in order to discover any interpretable patterns or trends with respect to the study question. In essence, each study included in a descriptive review is treated as the unit of analysis and the published literature as a whole provides a database from which the authors attempt to draw overall conclusions about the study question [8]. Authors of descriptive reviews extract from each study certain characteristics of interest, such as publication year, research methods, data collection techniques and direction of research outcomes (e.g., positive, negative, or non-determined) in the form of frequency analysis to produce quantitative results [9]. We used PubMed, Science Direct, Scopus, Web of Science and Google Scholar to collect the available research papers about foodborne infections in various food types throughout the Arab World in the past twenty years. We also searched for resources such as electronically accessible postgraduate theses and national reports that include details about foodborne pathogens in the region. The regional context of the Arab World consists of 22 countries in the MENA area, and these are: Algeria, Bahrain, Comoros, Djibouti, Egypt, Iraq, Jordan, Kuwait, Lebanon, Libya, Mauritania, Morocco, Oman, Palestine, Qatar, Saudi Arabia, Somalia, Sudan, Syria, Tunisia, United Arab Emirates and Yemen. These countries occupy an area extending from the Atlantic Ocean to the Zagros Mountains in southwest Asia. The Arab World occupies 14,291,469 km^2^, which comprises approximately 10.2% of the World’s landmass. Out of this, 72.5% is located in Africa and 27.5% in Asia. Around 90% of the Arab region consists of arid, semi-arid and dry sub-humid areas. The region is well known for its harsh environment and limited water resources and arable lands.

In this descriptive review we aim to elucidate (i) background knowledge and (ii) recent updates, based on published research in the past twenty years, on the prevalence of the three concerned pathogens (Non-Typhoidal *Salmonella*, *Campylobacter* and *Listeria*) in the food chain in the Arab countries. As a secondary objective, we also shed light on the reported evidence on the status of antimicrobial resistance (AMR) among the concerned three pathogens.

## 2. Non-Typhoidal *Salmonella* in the Food Chain in the Arab World

Non-Typhoidal *Salmonella* (NTS) are Gram-negative, rod-shaped bacteria within the family *Enterobacteriaceae*. NTS are among the most common foodborne zoonotic pathogens affecting humans worldwide [10,11]. These microbes are mostly transmitted to humans by ingesting contaminated food of animal origin, mainly beef meat, poultry meat, pork, milk, eggs, and contact with animals, including reptiles and turtles [1,12]. Nevertheless, other foods, including vegetables and fruits contaminated with animal feces, have also been involved in salmonellosis outbreaks in humans [13]. NTS usually causes mild-to-moderate self-limiting gastroenteritis, characterized by abdominal, diarrhea, vomiting, cramps, fever, and nausea but serious disease resulting in death does occur [6]. With recurrent infection, the NTS can spread beyond the intestine, giving rise to focal or invasive infections, mainly in immunocompromised patients, the elderly, or children. Ciprofloxacin and extended-spectrum cephalosporins are the most frequently used antibiotics for treating invasive salmonellosis infections in humans, especially children and the elderly [14]. Over the past decades, the increase of multidrug-resistant (MDR) forms of *S*. Enterica (*Salmonella* enterica subsp. enterica serotype Enterica, shortly *Salmonella* Enterica) in food-producing animals has been an emerging problem worldwide, likely due to the widespread use of common antimicrobials in poultry and animal husbandry for therapeutics, prophylaxis and growth promotion [12].

Measuring the prevalence of NTS in food products is important to quantify the risk of foodborne *Salmonella* infections in humans in the Arab World. According to a recent meta-analysis study by Rifai et al. [15], the pooled prevalence of NTS in the human population in the Arab countries was estimated at 6.6% (95% confidence interval (CI): 5.4–7.9%). In 2019 [15], Rifai et al. conducted a systematic review and meta-analysis to summarize current data on national and regional specific NTS prevalence in foods in the MENA region. The pooled prevalence of NTS was 8.8% (95% CI: 7.0–10.8%). The pooled prevalence of NTS in animal-sourced foods was 9.0% (95% CI: 6.8–11.4) based on studies from 13 countries. Furthermore, the pooled prevalence of NTS in fishery products and in plant-sourced foods (in six countries) was 22.9% (95% CI: 13.8–33.4%) and 0.4% (95% CI: 0.0–1.9%), respectively. Their study noted that *S*. Typhimurium (28.0%), S. Enteritidis (23.6%) and *S*. Kentucky (20.3%) were the most frequently reported serotypes in the tested food commodities in the MENA region. There is a scarcity of understanding of the magnitude of NTS in the food chain in the Arab countries. Additionally, little work has been done at the molecular characterization level to address the source-attribution of such an important pathogen [16].

Table 1 summarizes the published research depicting NTS prevalence rates in foods in the Arab World in recent years. In Algeria, Mezali and Hamdi [17] reported that 10% of the tested bovine carcasses (*n* = 190) and 12.7% (32/251) of ovine carcasses were found to be contaminated with *Salmonella*. In Egypt, in the Kafr El-Sheikh governorate, a study reported NTS occurrence in 4% (2/50) of the imported frozen beef liver [18]. Another study in Egypt detected higher rates of *Salmonella* at 60% (37/62) and 64% (14/20) in chicken parts and skin, respectively, whereas none of the tested table eggs samples (*n* = 60) were positive for *Salmonella* [19]. In Iraq, Harb et al. [16] reported the occurrence of *Salmonella* in 16% (16/100) of imported frozen chicken carcasses. In Morocco, according to Ibenyassine et al. [20], *Salmonella* was detected in 2% (1/50) of the sampled vegetables (Table 1). The results in Table 1 show a varying trend in the rates of *Salmonella* detection in various Arab countries, which might be attributed to the variability in the utilized laboratory methods, as well as variability in food sample types (animal-sourced versus plant-sourced) and their origin (local versus imported). Moreover, these results point to differences in *Salmonella* detection rates between studies from the same country; in Egypt, the contamination rate in chicken breasts varied widely between two studies [21,22], where in one study [21] the samples were from four northern cities, while in the other [22] samples were from one city in the south. Nonetheless, the collective descriptive results summarized in Table 1 affirm that NTS is present in food products consumed in Arab countries.

Given the abundant detection of NTS in various food commodities (Table 1), there is a need to strengthen national and regional surveillance efforts for antimicrobial resistance in NTS in our food chain [64]. In the primary production of animal-sourced foods, there has been an increase in the usage of the traditional first-line drugs sulfonamides, chloramphenicol, ampicillin, tetracycline and streptomycin and this has caused the emergence and development of resistant NTS due to selective pressure [65]. According to a study in Saudi Arabia by Badahdah [66], among 150 strains of *S.* Enteritidis that were isolated from local chickens, there was frequent resistance to streptomycin (55.8%), chloramphenicol (58.5%), neomycin (58.9%) and sulfamethoxazole (73.5%). In Egypt, according to Hassan et al. [67], 55% of *Salmonella* serotypes isolated from broilers showed resistance to nalidixic acid, and 89.3% were found resistant to tetracycline. Another study in Egypt investigated resistance in ten *Salmonella* strains isolated from poultry meat against amoxicillin-clavulanic acid (90%), trimethoprim-sulfamethoxazole (80%) and streptomycin, ampicillin-sulbactam, nalidixic acid, and gentamycin at 70% each [68]. In Morocco [58], 104 *S.* Enterica isolates from food origin were found resistant to tetracycline (21%), ampicillin (13%), amoxicillin-clavulanic acid (9%), streptomycin (7%), chloramphenicol (4%) and nalidixic acid (3.8%). Because NTS is associated with increased morbidity and mortality, the determination of antimicrobial resistance profiles should be regarded as a vital part of NTS surveillance in food safety and public health laboratories in Arab countries.

Implementation of evidence-based food safety systems in the Arab countries region will help better manage exposure to NTS—a step that will help lessen the public health burden of NTS infections in the region. NTS gastroenteritis’s global burden is estimated to be around 94 million infections, with a death estimate of 155 thousand and an average incidence rate of 1.14 episodes/100,000 inhabitants [69]. This reflects the disease’s enormous burden in both industrialized and developing countries [11]. For the WHO-defined East Mediterranean Region, the median incidence rate of NTS is 1610 illnesses, with 0.6 deaths per 100,000 persons [70]. The rate of salmonellosis incidence varies between countries across the Arab World and is influenced largely by the absence of systematic, harmonized national and regional surveillance and reporting systems. According to a recent systematic review and meta-analysis on prevalence of enteric NTS in humans in the Arab world, the highest pooled Salmonella prevalence measures were in Morocco (17.9%, 95% CI: 5.7–34.8%, 1997–2012), Tunisia (10.2%, 95% CI: 4.3–18.0%, 1988–2009) and Sudan (9.2%, 95% CI: 6.5–12.2%, 2006–2008), while the lowest were in Jordan (1.1%, 95% CI: 0.1–3.0%, 1993–2010), Oman (1.2%, 95% CI: 1.2%–1.3%, 1998–2002) and Palestine (1.2%, 95% CI: 0.4–2.1%, 1999–2011) [15]. Several published studies indicate that the most widely reported serovars associated with acute diarrheal disease across the Middle East are the *Salmonella serovars typhimurium* and *enteritidis* [71,72,73]. As the Middle East pattern, *S. typhimurium*, followed by *S. enteritidis*, has also been reported as the top-ranked serovars involved in human diarrheal illnesses across Africa, North America, and Oceania (Australia and New Zealand) [74]. On the other hand, *S. enteritidis* is more frequently reported than *S.* Typhimurium in human clinical isolates in Asia, Latin America, and Europe [75]. The variation in NTS serovars’ diversity among foods and humans is dynamic, and it is not surprising to capture variations between countries across the Arab World. Such variations could be explained by several factors impacting NTS levels in food, animals and the environment, which play a major role in human exposure to infection. Among these factors are food-animal production practices, climate, the environmental spread of specific serotypes, and the extent of vaccination programs in food animals. The public health burden of NTS in the Arab World and several published pieces of research in recent years (Table 1) stress the need to prioritize NTS in future preventative health plans in Arab countries.

## 3. *Campylobacter* in the Food Chain in the Arab World

The WHO report on the global burden of foodborne diseases concluded that foodborne transmission is considered the most important route for *Campylobacter* infection in the Middle East and globally [5]. *Campylobacter* spp. are microaerophilic, Gram-negative bacteria, spirally curved, motile rods with polar flagella, and intestinal commensals of mammals and birds [76,77]. They are slow-growing organisms that require selective media containing charcoal and various antibiotics, such as cephalothin, to suppress competing fecal microflora [78,79]. Microaerophilic bacteria require oxygen for growth but at lower levels than regular atmospheric air, and as such *Campylobacter* species grow best at 5–10% oxygen and 10% carbon dioxide [80,81]. *C. jejuni* and *C. coli* are thermotolerant (growing best at 42 °C) [78], while other *Campylobacter* species (such as *Campylobacter fetus*) are not [82]. *C. jejuni* and *C. coli* are the key cause of human campylobacteriosis, a very widely recognized foodborne illness that can be transmitted to humans through the consumption of improper cooked meat, especially chicken meat, contaminated milk and water and contact with farm animals such as poultry and livestock [83]. Human campylobacteriosis is characterized by watery or bloody diarrhea, abdominal pain, cramps, fever, malaise, and vomiting [84]. This illness is more dangerous in young children who are more prone to dehydration and loss of nutrients, such as sodium and protein, due to diarrheal illness [85]. Although antibiotics therapy is not generally indicated in most campylobacteriosis cases, treatment can decrease the duration and reduce the symptoms if initiated early in severe cases that warrant antimicrobial intervention [86]. Macrolides (specifical erythromycin) are considered the first choice of antimicrobials for the management of severe human *Campylobacter* infections [83].

Recent studies showed that *Campylobacter* spp. is widely presented in several food products in the Arab World [87,88,89], as described in Table 2. Much of the published research on *Campylobacter* in the Arab countries is centered around poultry (mainly chicken meat), followed by few studies in dairy products and other foods (Table 2). According to Ghaffoori [90], in Iraq, the prevalence of *C. jejuni* in broiler chicken carcasses (*n* = 20) was as high as 60%. In Saudi Arabia, *Campylobacter* Spp. was detected 5% (1/20) in retail unwashed eggshells that were sampled from retail at Al-Taif city [91]. In Tunisia, Jribi et al. [92] detected *Campylobacter* spp. in 23.7% of turkey meat samples (*n* = 101). In Egypt, *C. coli* was frequently isolated in 46.7% of chicken carcasses (*n* = 30) surveyed at Assiut city [93]. Other studies in Egypt isolated *Campylobacter* Spp. from raw milk [94,95]. Table 2 elaborates further on the recent studies on *Campylobacter* prevalence in various foods in the Middle East region. It is worth highlighting that there are very few studies on *Campylobacter* in the food chain in the GCC Countries. Such a gap in research in GCC countries should be considered urgently, as it is estimated that 2–28% of diarrheal cases in the Arabian Gulf countries are attributed to infection caused by *Campylobacter* [96]. Another critical gap is the lack in quantitative (counts/numbers) surveillance data for *Campylobacter* in the chicken meat supply across all Arab countries, despite the availability of several qualitative (presence/absence) surveillance data. The percentage of *Campylobacter*-contaminated poultry carcasses varies from country to country; it can be as low as 15%, as in Iceland, and as high as 90%, as in the USA [83]. Future research in the Arab World should conduct more quantitative surveillance, as this would form the base for developing quantitative risk assessment (QRA) and management plans for *Campylobacter* at national or regional levels. A few QRA pieces have been published regarding *Campylobacter* in the chicken meat supply across the Middle East [97].

According to the WHO, antimicrobial resistance in *Campylobacter* to erythromycin and fluoroquinolones has shown an increasing trend in many parts of the world [98]. Such increase appears to be associated with using these antimicrobials in poultry and livestock production systems [99,100]. In Egypt, the resistance of *C. jejuni* and *C. coli* isolates to ampicillin, streptomycin, chloramphenicol, erythromycin and tetracycline isolated from food of animal origin, especially poultry, proved to be more than 50% in isolates characterized in several studies [101,102]. According to a recent study in Iraq by Kanaan et al. [103], the rates of *C. jejuni* and *C. coli* toward tetracycline and erythromycin were up to 90%, yet lower rates of resistance were recognized toward enrofloxacin and gentamicin, of up to 30%. In Tunisia, Gharbi et al. [104] reported that 91 (100%) *C. jejuni* isolates from broiler chickens were resistant to erythromycin and tetracycline, and 90 (98.9%), 76 (83.5%), and 67 (73.6%) of the isolates were found resistance to ciprofloxacin, chloramphenicol and ampicillin, respectively. All *C. coli* isolates (*n* = 41) in the former study showed resistance to erythromycin, tetracycline, chloramphenicol and ciprofloxacin. In Morocco, Senok et al. [105] reported a high rate of antimicrobial resistance among *Campylobacter* isolated from chickens towards ciprofloxacin at 87.9% (29/33) and tetracycline at 78.8% (26/33). These results stress the need to investigate further the mechanism of antimicrobial resistance and the spread of resistance genes in *Campylobacter* sp. harboring food sources, notably chicken meat, in the Arab countries. The WHO declared *Campylobacter* as one of the 12 bacteria that pose the greatest threat to human health because of its resistance to antibiotics. In the United Arab Emirates (UAE), Sonnevend et al. [106] investigated the antibiotic sensitivity and the serotype and molecular type distribution of 41 *C. jejuni* strains isolated from diarrheal patients in a hospital in Al Ain (a major city in Abu Dhabi Emirate). While all strains were sensitive to erythromycin, 35 isolates (85.4%) exhibited resistance to ciprofloxacin [106]. These results point that the local incidence of fluoroquinolone resistance among *C. jejuni* was the highest reported worldwide. Infection with an antimicrobial-resistant *Campylobacter* strain may lead to a suboptimal antimicrobial treatment outcome or even treatment failure [107].

Although campylobacteriosis is a well-recognized problem in the Arab World countries, there are limited surveillance programs for *Campylobacter* in the region. Nationally representative prevalence and quantitative data of *Campylobacter* per gram of chicken are not currently available across the Arab World, notably the Arabian Gulf countries. Such data are required to fill a gap in assessing the microbial safety of chicken meat and could be used as an input for further development of QRA of *Campylobacter* in the Middle East.

## 4. *Listeria* spp. and *Listeria monocytogenes* in the Food Chain in the Arab World

Foodborne diseases caused by *Listeria monocytogenes* are a severe public health problem worldwide and in the Middle East. Contamination of fresh produce and animal-origin food with *L. monocytogenes* could occur at primary production or post-processing stages [91]. *L. monocytogenes* and *Listeria* spp. are a group of Gram-positive bacteria, non-sporulating rods with no capsule, facultative anaerobic and motile at 10–25 °C [118,119]. *Listeria* can multiply at a broad range of temperatures (0–45 °C) and pH (pH 4.5–9), and high salt concentrations (10% NaCl) [120,121]. The *Listeria* spp. consists of 17 species; however, only *L. monocytogenes* causes foodborne disease in humans, and *L. ivanovii* is only associated with animal infection. *Listeria* has been isolated from an assortment of food origins, including meat, seafood, dairy products, vegetables, water, plants, soil and feces of asymptomatic human and animal carriers [122,123]. Globally, listeriosis cause approximately 23,150 sicknesses per year and a fatality rate of 5463 deaths per year; listeriosis can be severe among the elderly, pregnant women and immunocompromised persons [124,125]. Consumption of contaminated food products accounts for an estimated 99% of all human listeriosis cases [126]. Clinical symptoms of listeriosis manifest as septicemia, meningitis, meningoencephalitis, prenatal infection, abortion and gastroenteritis [127,128]. *L. monocytogenes* infections are accountable for the highest hospitalization rates among all foodborne pathogens and have also been linked to large outbreaks of human illness worldwide [129,130].

*L. monocytogenes* has been isolated from food of animal origin and food of non-animal origin across the Arab countries. Several studies (Table 3) have reported *L. monocytogenes* in North African countries; however, few studies have been conducted in the GCC Countries. In Egypt, meat and meat products focus on several studies on the detection of *L. monocytogenes* from food animals, slaughterhouses, supermarkets and rural areas [131,132,133]. In Morocco, *L. monocytogenes* were frequently detected in raw milk and meat [134,135]. In Sudan, Alsheikh et al. [136] isolated *L. monocytogenes* from ready-to-eat chicken meat products in Khartoum restaurants. Yehia et al. [137] detected *L. monocytogenes* in raw beef, raw chicken, raw fish and raw camel milk from the wholesale poultry market located in Riyadh city in Table 3. While there are several reports on *L. monocytogenes* in animal-sourced foods, fewer are published on *L. monocytogenes* in plant-sourced foods in the Arab countries.

Amajoud et al. [134] reported a high occurrence of *L. innocua* (25%) and absence of *L. monocytogenes* in green salads in Tetouan, Morocco. Mohamed et al. [138] reported the occurrence of MDR *L. monocytogenes* at 14.2% (47/331) in vegetables (okra, carrot, green beans, artichoke, Molokhia, spinach, green peas, strawberry, grape leaves, broad bean, broccoli, grape, peach, salad, mixed vegetables, pomegranates and cauliflowers) purchased from various local stands in Egyptian markets. Despite the availability of some data on the prevalence of *L. monocytogenes* in retail foods in the Arab World, information on the serotypes and strains circulating in the region is limited.

Identification of possible pathways of *L. monocytogenes* and their antimicrobial resistance profile throughout the food chain, from farm-to-fork, will be of added value to the risk management of human illnesses in the Arab World [139]. In Jordan, Osaili et al. [140] detected antimicrobial resistance in *L. monocytogenes* isolates (*n* = 51) toward tetracycline and tilmicosin (9.8% each) in ready-to-eat chicken products. A recent study in Egypt by Aziz and Mohamed [141] reported nine *L. monocytogenes* isolates from meat with high resistance to penicillin, ampicillin, and tetracycline. The isolates in that study also showed resistance to erythromycin (66.6%), amoxicillin-clavulanic (55.5%), vancomycin (22.2%) and sulfamethoxazole/trimethoprim (11.1%). The high level of resistance to ampicillin is a real cause for concern considering that ampicillin is the drug of choice in empirical listeriosis therapy. Added to that, the occurrence of MDR *L. monocytogenes* isolates is an issue of public health concern that stresses the necessity for surveillance programs on antibiotic resistance evolution at the human-food interface. Food contamination by *L. monocytogenes* is not uncommon in the Arab World. Although considered a rare clinical pathogen, this microorganism has a very high mortality rate, particularly among infants, children and the elderly. Outbreaks of sporadic listeriosis cases have serious public health and economic implications that need to be curtailed through effective food safety management systems.

Some studies have been conducted in the Arab countries on *L. monocytogenes* at the human–food animal interface, notably in high-risk human groups. In Egypt, El-Gohary [142] detected *Listeria* spp. in 11.1% (1/9) of stool samples from women who had an abortion in the Mansoura governorate. EL-Naenaeey et al. [132] also isolated *L. monocytogenes* from 4% (8/200) of stools from healthy pregnant Egyptian women. Their study detected *inl*A and *inl*B genes in all isolates, indicating the potential virulence of such isolates. In Tunisia, a study by Elbeldi et al. [143] reported seven cases of infection with *L. monocytogenes* in Tunis city between 2000 to 2008; these were reported in two infants and five newborns children. The two infants’ clinical signs were fever associated with neurological and digestive signs, while maternal-fetal infections were confirmed in the cases reported in newborn children. In Algeria, Ramdani-Bouguessa and Rahal [144] report two listeriosis cases in newborns based on a single site (same hospital) investigation. In Morocco, there is a high public health vigilance to listeriosis in pregnant women; thus, for any case where a pregnant woman to be hospitalized with signs of a bacterial infection or unknown infection, *L. monocytogenes* infection should be suspected [145]. There is a need for strengthening surveillance systems of foodborne zoonoses in general, and for *L. monocytogenes* in particular, in the Arab countries. National food safety agencies should urge risk-based microbiological standards and strike a balance between meeting realistic and achievable limits for the industry while ensuring adequate protection. Individual countries across the Middle East should also realize the importance of national surveillance and audit existing food testing facilities to identify their limitations and improve their workflow to be up to far with international norms.

## 5. Conclusions

Foodborne infections caused by bacterial pathogens are a common cause of human illness in the Arab World, with a substantial burden resulting in economic loss and public health consequences. Non-typhoidal *Salmonella*, *Campylobacter* and *L. monocytogenes* have been frequently detected in various food types in Arab countries. The current evidence presented in this review points out that the detection of these bacterial pathogens is frequent in foods of animal origins. In fruit and vegetables, the available data on these pathogens are very modest compared to foods of animal origin. These bacteria may access the human food chain from primary production up to products’ final consumption. These bacterial pathogens are currently a significant concern for public health due to the emergence of multidrug-resistant strains. Despite reports on the prevalence of foodborne bacteria in foods of animal origin, livestock, and humans, the burden of these pathogens in foods of animal origin is still not well studied in the Arab region; the associated risk factors are not clearly identified, and the incidence of human infections from foodborne exposure is not well documented. This review’s literature suggests that *Salmonella*, *Campylobacter* and *L. monocytogenes* continue to challenge food safety and public health in the Arab World. Thus, we recommend the following: (1) A coordinated surveillance and monitoring system for foodborne pathogens should be established to design informed control and prevention strategies against these pathogens at national and regional levels across the Arab countries; (2) The epidemiological information regarding risk factors and incidence of human infections associated with foodborne infection should be established and well documented at the national level; (3) Public awareness should be developed based on pieces of evidence stemmed from a scientific risk analysis of bacterial pathogens causing foodborne infection; (4) It is crucial to utilize state-of-art molecular level characterization (e.g., whole-genome sequencing) to inform the implementation of better prevention and control strategies across the Arab countries.

## Figures and Tables

**Table 1:** The occurrence of Non-typhoidal *Salmonella* (NTS) in foods in the Arab countries.

Country	Tested Food Samples (Total Number)	% (Out of Total Number) of NTS Positive Samples	*Salmonella* Serotypes (%) *	References
Algeria	Frozen beef liver (*n* = 50)	4	ND	[18]
	Chicken Liver (*n* = 25)	4	Kentucky (100)	[23]
	Fruit and vegetable (*n* = 181)	ND (not detected)	ND	[24]
	Eggs (*n* = 45)	4.4	Bradford (100), Entritidis (0)	[25]
	Dairy products (*n* = 310)	ND	ND	[26]
	Raw red meat and meat products (*n* = 144)	23.6	Agona (1.6), Albany (3.1), Altona (12.5), Anatum (14.0), Corvallis (7.8), Enteritidis (7.8), Hadar (1.6), Heidelberg (4.7), Indiana (4.7), Infantis (1.6), Kedougou (1.6), Lexington (1.6), Liverpool (1.6), Mbandaka (4.7) Montevideo (6.3).	[17]
Egypt	Fresh poultry (*n* = 60)	10	Enteritidis (13.3), Typhimurium (60.0), Kentucky (6.7).	[27]
	Frozen poultry (*n* = 30)	3.3		
	Fresh beef (*n* = 60)	11.7		
	Frozen beef (*n* = 30)	3.3		
	Fresh vegetables and ready-to-eat salads (*n* = 121)	ND	ND	[28]
	Raw chicken meat (*n* = 100)	5	Typhimurium (60.0), Enteritidis (40.0).	[29]
	Poultry products (*n* = 75)	6.6	Enteritidis (40), Typhimurium (40), Kentucky (20)	[30]
	Fresh chicken meat (*n* = 200)	3.5	Enteritidis (14.3), Typhimurium (71.4), Kentucky (14.3).	[31]
	Ready-to-eat chicken meat (*n* = 100)	ND		
	Raw egg yolk (*n* = 30)	ND	Enteritidis (3.1), Typhimurium (0), Kentucky (41.5), Other types (55.4)	[19]
	Eggshell (*n* = 30)	ND		
	Mixed chicken meat samples (*n* = 62)	60		
	Chicken skin (*n* = 22)	64		
	Chicken carcasses (*n* = 50)	16	Enteritidis (37.4), Typhimurium (30.1), Kentucky (10.8), Muenster (8.4), Virchow (4.8), Anatum (4.8), Haifa (1.2), Other types (2.4)	[32]
	Frozen beef (*n* = 160)	2.5	Enteritidis (32.1), Typhimurium (41.5), Infantis (20.8)	[21]
	Fresh beef (*n* = 80)	18.7		
	Beef carcasses (*n* = 240)	8.3		
	Chicken breast (*n* = 160)	1.25		
	Chicken legs (*n* = 160)	7.5		
	Raw milk (Buffalo) (*n* = 240)	3.3		
	Raw milk (Cow) (*n* = 240)	1.6		
	Cheese (Kareish) (*n* = 120)	2.5		
	Cheese (Domiati) (*n* = 120)	0.83		
	Yogurt (*n* = 80)	ND		
	Frozen chicken breast fillets (*n* = 25)	52	Enteritidis (100)	[22]
	Frozen chicken legs (*n* = 25)	36	Enteritidis (100)	
	Minced frozen meats (*n* = 25)	20	Kentucky (100)	
	Frozen chicken (*n* = 100)	16	Enteritidis (12.5), Typhimurium (50.0), Kentucky (6.3), Newport (6.3), Muenchen (6.3), Hadar (12.5)	[16]
	Uncooked Hamburger (*n* = 10)	50	ND	[33]
	Fresh sheep and beef meat (*n* = 50)	4.5	ND	[34]
Jordan	Eggs (*n* = 25)	12	ND	[35]
	Fresh fish from Yemen (*n* = 110)	6.4	ND	[36]
	Pre-sliced imported cheddar cheese (*n* = 100)	10	ND	[37]
	Chicken Shawarma (*n* = 301)	1	ND	[38]
	Roasted chicken (*n* = 157)	0.6		
	Chicken burgers (*n* = 20)	ND		
	Beef kubba (*n* = 115)	0.9		
	Chicken Shawarma (*n* = 80)	13.8	Choleraesuis (100)	[39]
	Beef Shawarma (*n* = 20)	5		
	Raw milk (*n* = 20)	ND	ND	[40]
	White soft cheeses fresh (*n* = 20)	ND	ND	
	Labaneh (*n* = 20)	ND	ND	
	Fresh and frozen beef, lamb, and poultry meat (*n* = 300)	31	ND	[41]
Lebanon	Qishta’a (heat-coagulated milk) (*n* = 31)	42	ND	[42]
Morocco	Traditional cheese (*n* = 51)	5.9	Typhimurium (6.2), Enteritidis (4.2), Kentucky (22.9) Montevideo (6.2), Agona (16.7), Reading (12.5), Corvallis (8.3), Saintpaul (8.3) Israel (2.0), Hadar (2.0), Branderup (2.0)	[43]
	Minced meat (*n* = 138)	12.3		
	Sausage (*n* = 20)	5		
	Chicken (*n* = 86)	20		
	Turkey (*n* = 17)	52.9		
	Milk and other derivatives (*n* = 152)	ND.		
	Turkey sausages (*n* = 60)	23.3	Typhimurium (5.9), Agona (2.9), Saintpaul (2.9), Mbandaka (11.8), Montevideo (8.8), Livingstone (2.9), Corvallis (23.5), Kentucky (17.6), Bovismorbificans (5.9), Anatum (2.9), Give (11.8), Muenster (2.9)	[44]
	Beef sausages (*n* = 60)	15		
	Artisanal sausages (*n* = 36)	30.6		
	Cereal products (*n* = 60)	ND	-	[45]
	Chicken meat, eggs, and visceral organs (*n* = 432)	0.7	Hadar (9.1), Corvallis (18.2), Mbandaka (18.2), Ouakam (18.2), Tm var. Cop (9.1), Virchow (18.2), Altona (9.1).	[46]
	Mussels (*n* = 279)	10	Kentucky (57.1), Blockley (42.9), Senftenberg (0)	[47]
	Cooked meat (*n* = 2952)	0.7	Anatum (3.8), Bareilley (1.0), Berta (1.9), Blokley (10.4), Brenderup (6.6), Bredeney (12.3), Enteritidis (2.8), Hadar (3.8), Infantis (23.8), Kiambu (5.7)	[48]
	Sausages (*n* = 2052)	0.1		
	Chicken meat (*n* = 1200)	0.4		
	Pastry (*n* = 2232)	0.2		
	Chopped meat (*n* = 196)	2.4		
	Sea food (*n* = 562)	1.8		
	Spices (*n* = 80)	1.3		
	Slaughterhouses: beef meat (*n* = 2122)	3.5	Labadi (1.9), MBandaka (7.6), Montevideo (3.8), Typhimurium (8.5), Salamae type II (1.0), Non-typeable (2.8)	
	Raw ground beef (*n* = 150)	2	Entritidis (33.3), Typhimurium (33.3).	[49]
	Fresh sausage (*n* = 100)	4	Anatum (33.3), Bareilly: (14.3)	
	Chicken breast, legs, liver, gizzard (*n* = 576)	10	Typhimurium (40.4), Newport (26.3), Montevideo (17.5), Heidelberg (15.8)	[50]
	Vegetable samples (*n* = 50)	2	Arizona (100)	[20]
Libya	Uncooked chicken burger (*n* = 56)	12.5	ND	[51]
	Cooked spiced chicken burger (*n* = 64)	1.6		
Palestine	Raw milk, yogurt (*n* = 155)	ND	ND	[52]
	Pasteurized milk (*n* = 235)	ND		
	Concentrated yogurt, salt (*n* = 170)	ND		
	Cheese and cooked cheese (*n* = 109)	ND		
Saudi Arabia	Fish from Thailand (*n* = 98)	44.9	ND	[53]
	Fish from Vietnam (*n* = 25)	52		
	Fish from Bahrain (*n* = 35)	31.4		
	Fish from India (*n* = 50)	28		
	Fish from Myanmar (*n* = 15)	46.7		
	Frozen meat and fresh meat (*n* = 60)	25	ND	[54]
	West region (*n* = 40)	ND	ND	[55]
	East region (*n* = 40)	20		
	North region (*n* = 40)	ND		
	South region (*n* = 40)	7.5		
	Center region (*n* = 40)	2.5		
	Fresh vegetables (*n* = 68)	ND	ND	[56]
	Chicken (*n* = 100)	1	Arizona (100)	[57]
	Coffee beans (*n* = 31)	ND	ND	[58]
	Raw milk (*n* = 16)	ND	ND	[59]
Sudan	Raw and cooked food (*n* = 370)	2.4	Enteritidis (22.2), Typhimurium (11.1), Livingstone (11.1), Agona (11.1), Blockley: (11.1), Molade (22.2), I:rough-O:I,z13:1,5 (11.1)	[60]
Tunisia	Cooked dishes (*n*=150)	21.3	Zanzibar (0.08), Kentucky (28.0), Manchester (12.0), Schwarzengrund (0.08), Bredeney (4.0) Altona (12.0), Anatum (20.0), Amsterdam (4.0), Orion (4.0)	[61]
	Raw milk (*n* = 93)	33.3		
	Dairy products (*n* = 22)	22.7		
	Vegetables salad (*n* = 70)	12.8		
	Seafood (*n* = 46)	23.9		
	Raw poultry meat including (*n* = 45)	60		
	Cakes (*n* = 41)	26.8		
	Salami and sausage (*n* = 20)	25		
	Raw red meat (*n* = 13)	35.5		
	Chicken carcasses (*n* = 50)	16	Enteritidis (100)	[62]
	Cuts of beef (*n* = 144)	29.8	Enteritidis (3.8), Typhimurium (35.0), Kentucky (17.5), Suberu (15.0), Newlands (8.8), Zanzibar (13.8), Orion (7.5), Neumuenster (1.3)	[63]
	Raw chicken (*n* = 60)	48.3		
	Portions of minced meat (*n* = 56)	10.7		
	Cuts of lamb (*n* = 33)	6		
	Merguez (sausages) (*n* = 10)	ND		
	Fish (*n* = 12)	ND		

* The percentage (%) of *Salmonella* serotypes is calculated from the positive samples (isolated target bacteria).

**Table 2:** The occurrence of *Campylobacter* spp. in foods in the Arab countries.

Country	Tested Food Samples (Total Number)	*Campylobacter* spp. % *	*C. jejuni* %	*C. coli* %	Other Species	References
Egypt	Broiler (*n* = 101)	16.8	ND	4	ND	[108]
	Slaughterhouses (*n* = 104)	24	ND	3.9	ND	
	Fresh chicken meat products (*n* = 30)	53.3	46.7	46.7	ND	[93]
	Frozen chicken meat products (*n* = 30)	53.3	46.7	40	ND	
	Chicken burger (*n* = 15)	ND	ND	ND	ND	
	Chicken nuggets (*n* = 15)	13.3	13.3	13.3	ND	
	Raw milk (*n* = 50)	22	20	20	ND	[94]
	Kareish cheese (*n* = 50)	34	14	14	ND	
	Yoghurt (*n* = 50)	18	8	8	ND	
	Skin (*n* = 39)	30.8	12.8	17.9	*C. lari* (17.9), *C. hyointestinal* (0)	[109]
	Thigh meat (*n* = 39)	38.5	17.9	20.5	*C. lari* (20.5), *C. hyointestinal* (1)	
	Breast meat (*n* = 39)	41	33.3	5.1	*C. lari* (5.1), *C. hyointestinal* (2.7)	
	Raw milk (*n* = 50)	ND	4	ND	ND	[95]
	Laban Rayeb (*n* = 25)	ND	ND	ND	ND	
	Stored Domiati cheese (*n* = 39)	ND	ND	ND	ND	
	Fresh Domiati cheese (*n* = 38)	ND	11	ND	ND	
	Zabady (*n* = 25)	ND	ND	ND	ND	
	Ras cheese (*n* = 25)	ND	ND	ND	ND	
	Kariesh cheese (*n* = 25)	ND	ND	ND	ND	
Iraq	Retail frozen chicken meat (*n* = 40)	75	25	50	ND	[103]
	Broiler chicken carcasses (*n* = 20)	ND	60	ND	ND	[90]
	Broiler carcasses (*n* = 280)	ND	49.6	ND	ND	[110]
Jordan	Broiler chicken carcasses (*n* = 177)	31.6	17	ND	ND	[111]
	Broiler chicken (*n* = 50)	ND	30	ND	ND	[112]
	Live and Dressed Chicken (*n* = 140)	40	ND	ND	ND	[113]
Morocco	Broiler (*n* = 105)	71.4	ND	40	ND	[114]
	Raw poultry meat (*n* = 50)	62	ND	ND	ND	[115]
Qatar	Chicken skin (400)	36.5	29.3	ND	ND	[116]
Saudi Arabia	Chicken product (*n* = 99)	ND	52.3		ND	[88]
	Unwashed eggshell (*n* = 20)	5	ND	ND	ND	[91]
Sudan	Goats (*n* = 336)	83	5.7	69.6	*C. upsaliensis* (3.9%), *C. fetus* (2.1%), *C. lari* (1.8%)	[117]
Tunisia	Broiler chickens (*n* = 590)	22.4	ND	ND	ND	[104]
	Chicken meat (*n* = 149)	26.8	16.1	3.4	ND	[92]
	Turkey meat (*n* = 101)	23.7	13.8	1.9	ND	

* The percentage (%) of *Campylobacter* species is calculated from the positive samples (isolated target bacteria).

**Table 3:** The occurrence of *Listeria* spp. in foods in the Arab countries.

Country	Tested Food Samples (Total Number)	*Listeria* spp. % *	*L. monocytogenes* %	Other *Listeria* Species %	References
Algeria	Cheese (*n* = 385)	ND	5.2	ND	[146]
	Raw milk (*n* = 42)	14.3	ND	*L. innocua* (4.4), *L. seeligeri* (2.2).	[147]
	Sausage (*n* = 45)	6.7	2.2	*L. innocua* (4.4), *L. seeligeri* (0).	
	Foods ready to eat (*n* = 227)	9.3	2.6	*L. innocua* (4.8), *L. ivanovii* (1.3), *L. welshimeri* (0.4).	[148]
Egypt	Soft cheese (*n* = 155)	14.2	4.5	*L. innocua* (9.7).	[149]
	Milk of dairy cows (*n* = 200)	19	4	*L. Ivanovii* (6), *L. innocua* (2), *L. grayi* (3), *L. Welshimeri* (4)	[132]
	Minced meat (*n* = 20)	50	15	*L. ivanovii* (10), *L. welshimeri* (5), *L. innocua* (20), *L. seeligeri* (0), *L. grayi* (0).	[150]
	Kofta (*n* = 20)	70	20	*L. ivanovii* (5), *L. welshimeri* (10), *L. innocua* (14), *L. seeligeri* (10), *L. grayi* (5).	
	Sausage (*n* = 20)	35	10	*L. ivanovii* (10), *L. welshimeri* (5), *L. innocua* (10), *L. seeligeri* (0), *L. grayi* (0).	
	Burger (*n* = 20)	60	15	*L. ivanovii* (15), *L. welshimeri* (10), *L. innocua* (10), *L. seeligeri* (5), *L. grayi* (5).	
	Luncheon (*n* = 20)	40	10	*L. ivanovii* (10), *L. welshimeri* (10), *L. innocua* (5), *L. seeligeri* (5), *L. grayi* (0).	
	Pasterma (*n* = 20)	25	5	*L. ivanovii* (10), *L. welshimeri* (0), *L. innocua* (5), *L. seeligeri* (5), *L. grayi* (0).	
	Vegetable (*n* = 331)	ND	14.2	ND	[138]
	Raw milk, milking equipment, and hand swabs (*n* = 300)	26.3	23	*L. innocua* (3.3)	[151]
	Beef burgers (*n* = 50)	ND	32	ND	[152]
	Minced meat (*n* = 25)	ND	4	ND	
	Luncheon (*n* = 25)	ND	4	ND	
	Raw milk (*n* = 51)	27.5	3.9	*L. ivanovii* (3.9), *L. innocua* (9.8), *L. seeligeri* (5.9), *L. grayi* (2), *L. welshimeri* (2).	[153]
	Kareish cheese (*n* = 51)	13.73	2	*L. ivanovii* (2), *L. innocua* (2), *L. seeligeri* (6.5), *L. grayi* (0), *L. welshimeri* (2).	
	Burger (*n* = 50)	8	ND	*L. ivanovii* (2), *L. innocua* (0), *L. seeligeri* (0), *L. grayi* (4). *L. welshimeri* (2).	
	Butter (*n* = 31)	6.5	ND	*L. ivanovii* (3.2), *L. innocua* (0), *L. seeligeri* (0), *L. grayi* (0), *L. welshimeri* (3.2).	
	Frozen lean beef (*n* = 30)	20	3.3	*L. ivanovii* (6.7), *L. grayi* (3.3), *L. innocua* (3.3), *L. Seeligeri* (3.3), *L. welshimeri* (0).	[133]
	Raw milk (*n* = 30)	13.3	ND	*L. ivanovii* (3.3), *L. grayi* (3.3), *L. innocua* (0), *L. Seeligeri* (3.3), *L. welshimeri* (3.3).	
	Minced frozen beef (*n* = 25)	32	4	*L. ivanovii* (0), *L. innocua* (28), *L. welshimeri* (0), *L. seeligeri* (0), *L. grayi* (0), *L. murrayi* (0).	[131]
	Luncheon (*n* = 25)	32	8	*L. ivanovii* (4), *L. innocua* (0), *L. welshimeri* (8), *L. seeligeri* (8), *L. grayi* (4), *L. murrayi* (0).	
	Frozen chicken leg (*n* = 25)	52	ND	*L. ivanovii* (12), *L. innocua* (8), *L. welshimeri* (8), *L. seeligeri* (0), *L. grayi* (16), *L. murrayi* (0).	
	Frozen chicken fillet (*n* = 25)	56	8	*L. ivanovii* (12), *L. innocua* (12), *L. welshimeri* (12), *L. seeligeri* (0), *L. grayi* (8), *L. murrayi* (4).	
Iraq	Red meat (*n* = 375)	ND	13.9	ND	[154]
	Frozen chicken (*n* = 309)	ND	8.7	ND	[155]
	Fresh red meat (*n* = 167)	ND	6	ND	
	Gallbladder of sheep (*n* = 150)	ND	4	ND	[156]
	Gallbladder of cattle (*n* = 150)	ND	1.3	ND	
	Cheese (*n* = 50)	ND	2	ND	[157]
	Minced meat (*n* = 50)	ND	14	ND	
	Frozen chicken (*n* = 50)	ND	8	ND	
Jordan	Raw and processed meat (*n* = 270)	ND	24.4	*L. ivanovii* (27), *L. grayi* (8.9), *L. seeligeri* (3.7), *L. welshimeri* (2.2), *L. innocua* (6.3)	[158]
	Ready-to-Eat Meat Products (*n* = 1028)	ND	2	ND	[38]
	White Cheese (*n* = 350)	27.1	11.1	*L. grayi* (6.9%), *L. innocua* (2%), *L. ivanovii* (4%), *L. seeligeri* (2%), and *L. welshimeri* (0.3%)	[159]
	RTE chicken-Shawirma (*n* = 30)	ND	13.3	*L. ivanovii* (66.7), *L. grayi* (6.6), *L. seeligeri* (3.3), *L. welshimeri* (0).	[140]
	Fresh broiler chicken (*n* = 160)	ND	9.4	*L. ivanovii* (30), *L. grayi* (5), *L. seeligeri* (2.5), *L. welshimeri* (0.6).	
	RTE chicken-burger (*n* = 30)	ND	76.7	*L. ivanovii* (10), *L. grayi* (0), *L. seeligeri* (0), *L. welshimeri* (0).	
	RTE chicken-sausage (*n* = 30)	ND	30	*L. ivanovii* (6.7), *L. grayi* (0), *L. seeligeri* (0), *L. welshimeri* (3.3).	
	Mortadella (*n* = 30)	ND	ND	*L. ivanovii* (0), *L. grayi* (0), *L. seeligeri* (0), *L. welshimeri* (0).	
	RTE beef meat (*n* = 120)	ND	19.2	*L. innocua* (24), *L. welshimeri* (14.2).	[160]
	Ready-to-eat poultry meat (*n* = 120)	ND	15	*L. innocua* (23), *L. welshimeri* (15.8).	
Libya	Raw cow milk (*n* = 20)	45	20	ND	[161]
	Laben (*n* = 20)	35	5	ND	
	Ricotta cheese (*n* = 20)	40	5	ND	
	Maassora cheese (*n* = 20)	40	5	ND	
	Chicken meat (*n* = 20)	45	20	ND	
	Chicken burger (*n* = 20)	60	15	ND	
	Raw beef (*n* = 20)	65	20	ND	
	Beef burger (*n* = 20)	60	5	ND	
	Beef sausage (*n* = 20)	55	20	ND	
Morocco	Beef meat (*n* = 140)	ND	7.1	ND	[162]
	Dairy products (*n* = 404)	3.2	0.7	*L. innocua* (1.5), *L. welshimeri* (0.7), *L. seeligeri* (0.3).	[134]
	Bovine meat products (*n* = 258)	12.8	2.7	*L. innocua* (9.3), *L. welshimeri* (0), *L. seeligeri* (0).	
	Poultry meat products (*n* = 103)	14.6	ND	*L. innocua* (13.6), *L. welshimeri* (1), *L. seeligeri* (0).	
	Pastries (*n* = 162)	4.9	3.1	*L. innocua* (1.9), *L. welshimeri* (0), *L. seeligeri* (0).	
	Salads (*n* = 143)	2.8	ND	*L. innocua* (2.8), *L. welshimeri* (0), *L. seeligeri* (0).	
	Chickpea flour cooked with eggs sold in the street (*n* = 20)	25	ND	*L. innocua* (25), *L. welshimeri* (0), *L. seeligeri* (0).	
	Mayonnaises (*n* = 6)	33.3	16.7	*L. innocua* (16.7), *L. welshimeri* (0), *L. seeligeri* (0).	
	Raw milk, Lben and Jben (*n* = 288)	ND	5.9	ND	[136]
Saudi Arabia	Raw beef (*n* = 10)	50	ND	*L. innocua* (10), *L. seeligeri* (10), *L. welshimeri* (20), *L. grayi* (10), *L. ivanovii* (0).	[91]
	Raw chicken (*n* = 10)	50	20	*L. innocua* (10), *L. seeligeri* (10), *L. welshimeri* (0), *L. grayi* (0), *L. ivanovii* (10).	
	Raw fish (*n* = 10)	30	ND	*L. innocua* (0), *L. seeligeri* (10), *L. welshimeri* (10), *L. grayi* (10), *L. ivanovii* (0).	
	Raw camel milk (*n* = 10)	20	ND	*L. innocua* (0), *L. seeligeri* (0), *L. welshimeri* (0), *L. grayi* (10), *L. ivanovii* (0).	
	Unwashed eggshell (*n* = 20)	10	ND	ND	[91]
Sudan	Raw milk (*n* = 120)	7.5	2.5	*L. innocua* (0), *L. Seeligeri* (1.7), *L. welshimeri* (1.7), *L. ivanovii* (1.7), *L. grayi* (0), *L. murrayi* (0).	[163]
	Frozen chicken meat (*n* = 500)	39	12.8	*L. ivanovii* (19.4), *L. grayi* (4), *L. seeligeri* (1), *L. welshimeri* (1.8).	[164]
Syria	Cow milk (*n* = 345)	70	6.7	*L. innocua* (3.2), *L. ivanovi* (2), *L. gravi* (0.9), *L. welshimeri* (1.4),	[165]
	Sheep milk (*n* = 225)	30	5.3	*L. innocua* (1.8), *L. ivanovi* (2.2), *L. gravi* (0.4), *L. welshimeri* (1.3),	
Tunisia	Fish (*n* = 50)	-	2	Not reported	[166]
	Sausage (*n* = 30)	-	3	Not reported	

* The percentage (%) of *Listeria* species is calculated from the positive samples (isolated target bacteria).

## Data Availability

All of the data used in this research appears in the manuscript and is available at request from corresponding author.
